# A Perspective on *Withania somnifera* Modulating Antitumor Immunity in Targeting Prostate Cancer

**DOI:** 10.1155/2021/9483433

**Published:** 2021-08-25

**Authors:** Seema Dubey, Manohar Singh, Ariel Nelson, Dev Karan

**Affiliations:** ^1^Department of Pathology, MCW Cancer Center and Prostate Cancer Center of Excellence, Medical College of Wisconsin, 8701 Watertown Plank Road, Milwaukee, WI 53226, USA; ^2^Department of Medicine, Division of Hematology and Oncology, Medical College of Wisconsin, 8701 Watertown Plank Road, Milwaukee, WI 53226, USA

## Abstract

Medicinal plants serve as a lead source of bioactive compounds and have been an integral part of day-to-day life in treating various disease conditions since ancient times. Withaferin A (WFA), a bioactive ingredient of *Withania somnifera*, has been used for health and medicinal purposes for its adaptogenic, anti-inflammatory, and anticancer properties long before the published literature came into existence. Nearly 25% of pharmaceutical drugs are derived from medicinal plants, classified as dietary supplements. The bioactive compounds in these supplements may serve as chemotherapeutic substances competent to inhibit or reverse the process of carcinogenesis. The role of WFA is appreciated to polarize tumor-suppressive Th1-type immune response inducing natural killer cell activity and may provide an opportunity to manipulate the tumor microenvironment at an early stage to inhibit tumor progression. This article signifies the cumulative information about the role of WFA in modulating antitumor immunity and its potential in targeting prostate cancer.

## 1. Introduction

Since ancient times, people have used herbal medicines from plants or their extracts derived from flowers, seeds, bark, leaves, or roots to prevent or treat multiple disease types. Despite significant progress in early detection, advancement in understanding the molecular targets, and improved anticancer therapy, prostate cancer remains the second most common male malignancy and the fifth leading cause of cancer deaths among men worldwide [1]. Many useful bioactive compounds, currently in use as chemotherapy drugs, including taxanes, the camptothecin derivatives, the epipodophyllotoxins, and the vinca alkaloids, have been extracted and isolated from plant sources [2]. In addition, epidemiological studies support that dietary modification may substantially reduce a man's risk of developing prostate cancer [3, 4]. Therefore, understanding the role of plant-based therapies in determining the immune mechanisms of prostate cancer prevention and the therapeutic efficacy is crucial.

*Withania somnifera*, also known as Indian ginseng, is a traditional Ayurvedic medicinal plant that contains diverse bioactive compounds, including alkaloids, withanolides, and saponins. The main phytochemicals are withanolides, which are structurally similar to the ginsenosides of *Panax ginseng* (Asian ginseng), *Panax notoginseng* (Sanchi ginseng), and *Panax quinquefolius* (American ginseng). However, the ginsenoside contents within the *Panax* species vary significantly [5]. The pharmacological activity of *W. somnifera* is assigned to its main withanolide—Withaferin A (WFA) [6]. Although various parts of this plant are used for multiple disease treatments, root extract is a rich source of WFA. Growing evidence has shown that WFA has anti-inflammatory, antimicrobial, and anticancer activities [7–10]. This review highlights the effect of WFA in regulating both the immunological and nonimmunological targets and its potential as an anticancer agent for prostate carcinoma.

## 2. Nonimmune Molecular Targets of WFA

Withaferin A is a nontoxic, bioactive compound of *Withania somnifera*, a widely used traditional medicine in Asia and Africa for its anticancer activities and to enhance the immunological response. Numerous studies have described the anticancer effect of WFA in various cancer types, including leukemia, melanoma, prostate cancer, breast cancer, ovarian cancer, head and neck cancer, and colon cancer [11–14]. WFA inhibits cell proliferation, invasion, metastasis, angiogenesis, proteasome, endoplasmic reticulum (ER) stress, protein folding, and maturation in cancer cells and regulates multiple targets by direct interaction or regulation of secondary targets in establishing its anticancer activity. Since several succinct review articles have described the role of WFA in various cancer types [11, 12, 15], we limit our discussion to prostate cancer along with important molecular targets from other cancer types.

### 2.1. WFA-Mediated Cell Cycle Inhibition

Analysis of cell cycle-related events is a primary mechanism to examine a natural compound's biological effect in targeting cancer cells. WFA inhibits cell proliferation by inducing cell cycle arrest in the G2/M phase in multiple studies [16–20]. Dysregulation in the cell cycle process is associated with prostate cancer development, and currently, several cell cycle inhibitors are being evaluated in clinical trials for prostate cancer treatment [21]. In prostate cancer cell lines PC3 and DU145, WFA showed cell cycle inhibition in the G2/M phase by upregulating p21, phosphorylated wee-1, phosphorylated histone H3, and aurora B and downregulating cyclin (A2, B1, and E2) expression [22]. Interestingly, WFA showed a higher cytotoxic effect in androgen-resistant, androgen receptor (AR) negative cell lines PC3 and DU145 than androgen-sensitive, AR positive LNCaP cells [23]. In addition, WFA increased prostate apoptosis response-4 protein (Par-4) to enhance proapoptotic signaling in prostate cancer cells [14].

Following other targeted mechanisms, WFA decreased cyclin-dependent kinase (Cdk1), cell division cycle (Cdc 25B), and Cdc 25C expression and arresting cell cycle in the G2/M phase in breast cancer [18]. WFA prevents Cdk1/cyclin B1 complex formation, a critical step of cell cycle progression in gastric cancer, by dephosphorylating Cdk1 at Thr161 and p21 upregulation in glioblastome [16]. Additional studies showed that increased oxidative stress is critical in the WFA-mediated cell cycle and cell proliferation inhibition [17]. WFA-directed reticence in cell proliferation and migration is credited to blocking STAT3 transcription activity in colon cancer [24]. More importantly, combined treatment of WFA and liposomal preparation of doxorubicin enhanced cell death in ovarian cancer cells and inhibited the expression of cancer stem cell markers ALDH1 (aldehyde dehydrogenase) and Notch1 [25]. WFA is also known to restore tumor necrosis factor-related apoptosis-inducing ligand (TRAIL) sensitivity inducing apoptosis in human renal and breast cancer cells [26, 27].

### 2.2. Proteasomal Inhibition by WFA

Ubiquitin proteasome plays a critical role in neoplastic cell growth, survival, and apoptosis by reducing unwanted and misfolded proteins. Proteasome inhibitors showed anticancer activity in cancer cells resistant to conventional chemotherapeutic drugs. Bortezomib was the first proteasome inhibitor approved to treat refractory multiple myeloma [28]. WFA treatment of cancer cells showed an accumulation of ubiquitinated proteins signifying its effect on proteasome inhibition, associated with increased ER stress, reactive oxygen species, and proapoptotic signaling [29, 30]. Ghosh et al. reported WFA-induced cell death in breast cancer cells by introducing impaired autophagy and unfolded protein response [31]. WFA inhibits chymotrypsin-like activity of a 26S proteasome in PC3 xenografts in nude mice and the PC3 cell line, leading to accretion of proteasome target proteins p27, Bax, and I*κ*B-*α* and increase in PARP cleavage proteins inducing apoptosis in prostate cancer cells [32]. Additional docking studies revealed that WFA blocks the chymotrypsin-like activity of a purified rabbit 20S proteasome activity by blocking the N-terminal threonine (Thr1) function.

### 2.3. WFA Inhibits Angiogenesis

Angiogenesis is one of the hallmarks of cancer growth, where vascular endothelial growth factor (VEGF) regulates angiogenesis. The utilization of anti-VEGF therapies has been successful in prolonging survival benefits in multiple cancer types. Increased VEGF expression in prostate tissues and plasma levels has been associated with tumor grade and biochemical and clinical recurrence of prostate cancer [33, 34]. Anti-VEGF therapy in phase II clinical trials in prostate cancer showed improved relapse-free survival and disease stabilization [35]. However, the phase III results of antiangiogenic treatment in prostate cancer showed toxicity without improving the overall survival [36]. Plant-based compounds are efficient in preventing the formation of new blood vessels in the tumor microenvironment; hence, several natural compounds such as curcumin, resveratrol, and thymoquinone have been studied as potential antiangiogenic drugs [37–40]. In human umbilical vein endothelial cells (HUVECs), WFA showed an antiangiogenic effect by inhibiting HUVEC sprouting in the three-dimensional collagen-I matrix [41]. Similarly, WFA deters VEGF-mediated tube formation by HUVECs and binds to vimentin and intermediate filament protein to inhibit tumor angiogenesis [42, 43].

### 2.4. Effect of WFA on Tumor Growth in Mice

In prostate mouse models (TRAMP: transgenic adenocarcinoma of mouse prostate and Pten-knockout) with spontaneous tumor development, oral administration of WFA showed a significant decrease in prostate tumor growth. TRAMP mouse studies revealed that WFA inhibited AKT and pAKT expression and activated Foxo3a-Par-4-induced tumor cell death in mice [44]. Foxo3a works upstream of Par-4 signaling and is essential for apoptosis induction in castration-resistant prostate cancer (CRPC) cells. Deleting the Foxo3a binding site on the Par-4 promoter inhibits Foxo3a-Par-4 interaction and Par-4 activation, suggesting the positive role of Foxo3a in Par-4 stimulation and apoptosis [45]. In Pten-knockout mice, WFA treatment obliterated lung metastasis of prostate cancer and was associated with a decrease in epithelial-to-mesenchymal transition markers (N-cadherin and *β*-catenin) [46]. A significant reduction in tumor growth with subcutaneous xenografts of ovarian cancer and glioblastoma cells in nude mice also demonstrated the therapeutic efficacy of WFA [47, 48].

Thus, WFA showed pleiotropic functions and exerted its potential anticancer effect via targeting multiple molecular pathways to incapacitate the cancer cell activities.  Table 1 summarizes the nonimmune molecular targets of WFA in prostate cancer.

## 3. Effect of WFA in Targeting Inflammation via Inflammasomes

The association of chronic inflammation in cancer development and progression is well-established [51–54]. Therefore, studies are focused on targeting inflammatory cytokines to understand the immunobiology of prostate cancer [55–58]. Mechanistically, a diverse array of signals such as inflammasomes, toll-like receptors (TLRs), and transcription factors regulate the secretion of inflammatory cytokines. Upregulated inflammasome activity is correlated with various cancer types, including gastric cancer, breast cancer, prostate cancer, and brain tumor [59–61]. TLRs, a family of pattern recognition receptors, drive inflammation via activating NF-*κ*B signaling to promote prostate cancer development [62, 63]. Indeed, the transcriptional activity of NF-*κ*B and associated signaling pathways are implicated in multiple disease types and have been a prime target for pharmacological interventions [64]. Nuclear translocation of NF-*κ*B in prostate cancer cells was associated with biochemical recurrence and bone metastatic prostate cancer development. In a retrospective study using a multi-institutional cohort, Grosset et al. showed that an increased level of nuclear NF-*κ*B p65 might serve as a prognostic marker for prostate cancer [65]. The study performed tissue microarrays of radical prostatectomy specimens from two independent cohorts (*n* = 250 and *n* = 1262) of treatment-naïve prostate cancer patients collected at multiple centers. The association of p65 nuclear expression and prostate cancer outcome was evaluated by immunohistochemistry. Multivariate analysis revealed that p65 nuclear frequency in prostate cancer cells was an independent predictor of biochemical recurrence and may help identify high-risk prostate cancer patients. In another study, a similar analysis of NK-*κ*B p65 in 105 prostate tissue specimens showed nuclear translocation of p65 expression during the transition of disease from prostatic intraepithelial neoplasia to prostate cancer [66]. Multivariate analysis showed that preoperative PSA level, Gleason score, and nuclear KF-*κ*B p65 translocation were independent predictors of biochemical relapse. Thus, blocking NF-*κ*B activation is a promising target for modifying the cancer-associated inflammation and immune activation signaling.

Dietary agents are considered potent inhibitors of the NF-*κ*B signaling pathway and reduce cancer-associated inflammation [67]. For example, curcumin, a dietary supplement from turmeric spice, and ginger extract showed anticancer and anti-inflammatory effects by inhibiting NF-*κ*B activity [68–71]. Lycopene blocks NF-*κ*B signaling to inhibit prostate cancer cell growth in vitro and reduces in vivo prostate cancer growth in mice [72, 73]. In a chronic prostatitis rat model, the intragastric lycopene administration (20 mg/kg, daily for four weeks) reduces inflammation in the prostate and the serum level of IL-1*β*, TNF-*α*, IL-2, and IL-6 cytokines [74]. This anti-inflammatory effect of lycopene was attributed to NF-*κ*B inhibition in the prostate tissues [74]. Resveratrol isolated from grapes is also one of the potent inhibitors of NF-*κ*B and leads to anti-inflammatory and antitumor effects [75]. Likewise, ursolic acid derived from berries, leaves, and fruits is a pentacyclic triterpenoid with anti-inflammatory effects by inhibiting NF-*κ*B signaling through IKK*α* and p65 phosphorylation [76]. WFA inhibits NF-*κ*B activation by blocking Akt to reduce nitric oxide and inducible nitric oxide synthase expression in RAW 264.7 macrophage cell line regulating inflammatory process [77], suggesting that WFA has alternative pathways to regulate NF-*κ*B activation. Based on protein array analysis, it has been demonstrated that WFA regulates multiple cytokines in LPS-induced THP-1 cells. In silico studies showed that the downregulated cytokines possess a common regulatory factor NF-*κ*B and that WFA blocked the nuclear translocation of NF-*κ*B [78]. The root extract of *W. somnifera* is also known for its antioxidant, anti-inflammatory, and immunomodulatory activities [79, 80]. Culture supernatant from the LPS-primed, *W. somnifera* root extract- (0.05-0.4 mg/ml) treated human PBMCs, and THP-1 cells showed decreased TNF-*α*, IL-1*β*, and IL-6 levels as measured by ELISA [79]. However, further discussions are limited in this article since a recent review described a general perspective of WFA in chronic inflammation [81]. These studies suggest that the medicinal plant product regulating NF-*κ*B activity can alter inflammatory profiles to benefit the host, targeting inflammation-associated cancer.

Scientific advancement in understanding the regulation of inflammation also suggests the role of the inflammasome signaling complex in the activation of inflammation-associated responses. The inflammasome consists of a nucleotide-binding domain, leucine-rich repeat, and pyrin domain (NRLP), along with caspase activation and recruitment domain (ASC) and procaspase-1. Microbe-associated molecular patterns (MAMPs), danger-associated molecular patterns (DAMPs), pattern-recognition receptors (PRRs), and various pathogens activate the inflammasome. Once activated, the inflammasome recruits the ASC and procaspase-1 producing an active form of caspase-1, which cleaves the proform of interleukin- (IL-) 1*β* and IL-18 to release mature IL-1*β* and IL-18 associated with various biological activities [60, 61].

Although limited information is available on the effect of WFA in regulating inflammasomes, a mouse model of chronic pancreatitis showed that WFA blocks ER stress and the NLRP3 inflammasome to prevent pancreatitis progression [82]. WFA inhibits *Helicobacter pylori*-induced IL-1*β* and NLRP3 inflammasome signaling molecules in bone marrow-derived dendritic cells and macrophages, indicating the preventive and therapeutic effect of WFA in *H. pylori* infection-associated cancers [83]. Pretreatment of mice with WFA inhibited ovalbumin-induced lung injury and fibrosis progression. This preventive effect was attributed to decreased inflammatory cell infiltration in the lungs and a correspondingly low level of proinflammatory cytokines and reduced NLRP3 inflammasome activation [84]. WFA disintegrates the NLRP3 inflammasome complex reducing downstream signaling products IL-1*β* and IL-18. WFA blocks the NF-*κ*B activity altering the level of multiple genes associated with NF-*κ*B regulation and inhibits inflammasome activity suppressing inflammatory cytokine network [78]. Growing evidence suggests that the inflammasomes play a key role in inflammation-associated diseases, including cancer, and emerges as a game-changer in understanding how inflammation affects the immunobiology of cancer [61]. Therefore, the mechanism of WFA in the regulation of inflammasome activity warrants further investigation.

## 4. Effect of WFA on Immune Cell Regulation and Antitumor Immunity

Medicinal herbs have long been recognized as a way to increase immune system activation. However, the mechanism(s) regulating specific immune cells' functional activation by these herbs, including WFA, remains mostly unknown. Barua et al. showed that *W. somnifera* treatment induces natural killer (NK) cell activation [85], an essential component of the innate immune response to tumors, and actively eliminates early neoplastic cells. NK cells kill tumor targets on contact using perforin (a cytolytic protein) and granzyme (protease family member) machinery [86, 87]. Similarly, IFN-*γ* is a key cytokine produced by NK cells and T lymphocytes and facilitates the antitumor response. Malik et al. showed that herbal formulation of *W. somnifera* induced Th1 immunity in tumor-bearing mice as measured by the secretion of IFN-*γ* and IL-12 and increased proliferation of CD4^+^, CD8^+^ T cells, and NK cells [88]. Following *W. somnifera* supplement, hens susceptible to ovarian cancer displayed a significant reduction in tumor development associated with increased NK cell population [85].

Similarly, oral administration of *W. somnifera* extract (400 mg/kg body weight) once a week for four weeks treating azoxymethane-induced colon cancer in Swiss albino mice increased the number and functional activity of immune cells [89]. NMITLI 101R, a chemotype of *W. somnifera*, generated humoral and cellular immune responses in Balb/c mice as measured by a high number of antibody-producing cells. The cytokine response remained polarized to the Th1 type with increased levels of IFN-*γ* [90]. In parasite-infected hamsters, the chemotype NMITLI 101R surges antileishmanial drug efficacy by generating strong IFN-*γ*- and IL-12-mediated immune responses while suppressing the Th2 cytokines (IL-4, IL-10, and TGF-*β*) [91].

**Figure 1:**
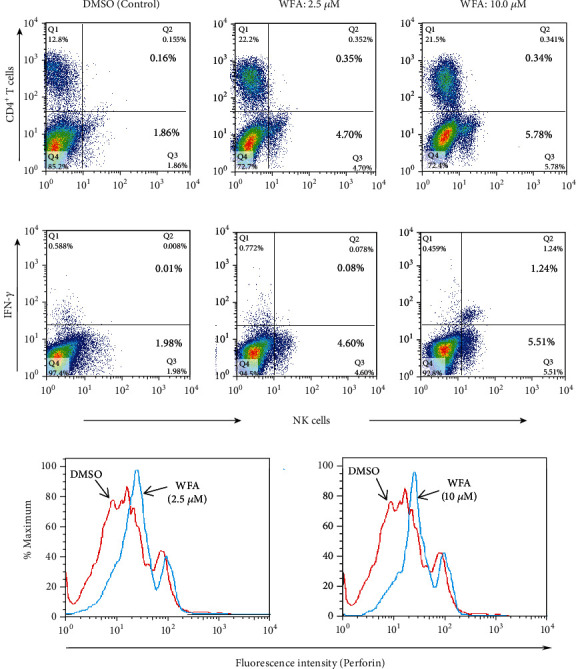
(a) WFA-induced frequency and (b) function (increase in IFN-*γ* and perforin) of NK cells examined by flow cytometry gated on CD8-negative T cells. Splenocytes from the naïve C57BL/6 mice were homogenized into a single-cell suspension, seeded at a density of 5 × 10^6^ cells/well supplemented with cytokine IL-2 (10 U/ml) in 24-well plates, and incubated overnight in the presence or absence of WFA and DMSO as control.

The present team also examined the effect of WFA and found that the WFA augments the quality and quantity of NK cell function measured by intracellular cytokine staining for IFN-*γ* and perforin. The number of NK cells producing IFN-*γ* and perforin was significantly higher in WFA-treated splenocytes than in the DMSO control (Figures 1(a) and 1(b)).

**Figure 2:**
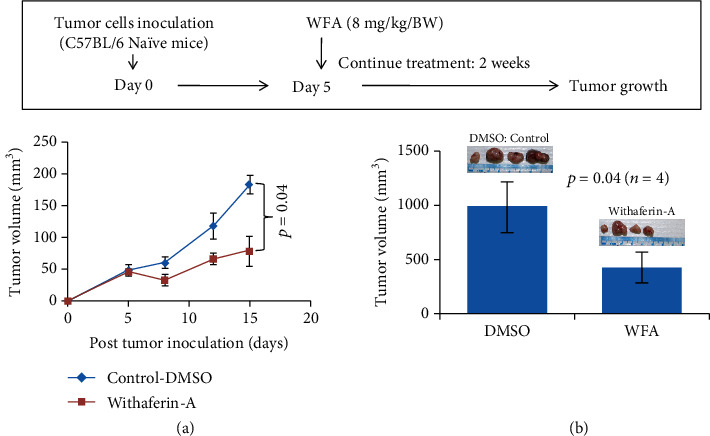
(a) WFA-induced activation of anticancer activity inhibiting the growth of established tumors in mice inoculated with RM1 mouse prostate tumor cells; (b) tumor volume on day 29. Six-to-seven-week-old, naive C57BL/6 mice were inoculated subcutaneously with RM1 tumor cells (5 × 10^5^). Starting on day five following tumor inoculations, a group of mice (4 mice/group) received an intraperitoneal (i.p.) injection of WFA (8 mg/kg/BW) five days per week for two weeks. DMSO serves as the control at the same level (*v*/*v*) as for WFA treatment groups.

To further study the role of WFA-induced NK cells in antitumor immunity, the authors used RM1 mouse prostate tumor cells (syngeneic to C57BL/6 mice). RM1 cells express a low level of MHC I and are sensitive to NK cell killing [92]. Intraperitoneal administration of WFA (8 mg/kg/BW) significantly (*p* = 0.04) inhibited the growth of established prostate tumors (Figures 2(a) and 2(b)). To follow the prolonged therapeutic effect of WFA, these experimental mice were left untreated for ten additional days. It appeared that the developed immune response following WFA treatment maintained the reduced tumor growth (Figure 2(b)). These observations indicate that the WFA induces NK cell activation, thereby augmenting antitumor immunity to inhibit the development and progression of prostate tumors; however, these preliminary studies need further validation.

Both mouse and human studies further support the role of WFA in the induction of NK cells. Balb/c mice were given an i.p. injection of *W. somnifera* root extract with 20 mg/dose/mouse for 5 consecutive days [93]. Mouse splenocytes were harvested 24 hours after the last injection, and were used as effectors against NK-sensitive K562 cells in a chromium release assay to measure the cytotoxic activity of NK cells. Splenocytes from WFA-treated mice showed ~53% higher cytotoxicity than splenocytes from vehicle-treated control mice. An observational study in humans also supports WFA-mediated NK cell regulation. Five human subjects consumed 6 ml of *W. somnifera* root extract with cow's milk (8 ounces) twice daily for four days. Peripheral blood sample analysis revealed a significant increase in mean fluorescence intensity of CD4^+^ T (4.2-fold; *p* < 0.05) cells and NK cell activation (3.2-fold; *p* < 0.01) after four days compared to the baseline [94].

Although therapeutic targeting of prostate cancer has evolved remarkably, one significant challenge remains that prostate tumors are enriched with immune suppressor cells (myeloid-derived suppressor cells (MDSCs), M2 macrophages, and T-regulatory cells), hampering the benefits of therapeutic regimens [95, 96]. Sinha and Ostrand-Rosenberg reported that administration of WFA in tumor-bearing mice with 4T1 cells significantly reduces the tumor burden, decreases the number of MDSCs and reactive oxygen species (ROS), and suppresses the protumor cytokine IL-10 by the MDSCs and macrophages [97]. WFA was orally administered in mice at different doses (1-8 mg/kg body weight) every other day for the study duration and found that 1 mg/kg dose of WFA was efficient in delaying tumor growth and suppressing MDSCs in the circulation. In vitro analysis revealed that 1 *μ*g/ml of WFA reduces ROS (measured by H_2_O_2_) production by >50% in MDSCs while 1 *μ*g/ml treatment dose of withanolide A and *W. somnifera* root extract was ineffective. The dose of *W. somnifera* root extract was increased to 166.7 *μ*g/ml to achieve a concentration equal to 1 *μ*g/ml of WFA for complete inhibition of H_2_O_2_ production in MDSCs. Withaferin A also inhibited the secretion of proinflammatory cytokines TNF-*α*, IL-6, and IL-12 from macrophages suggesting the role of WFA as a potent inhibitor of proinflammatory mediators [97]. This study corroborates with previous annotations that the use of WFA polarized the immunity towards the Th1 type augmenting antitumor immunity [88, 90, 91]. Thus, the stimulation of NK cell activity and decreased immune suppressor cells provide a novel opportunity to test the immunotherapeutics of WFA in prostate cancer studies.

**Figure 3:**
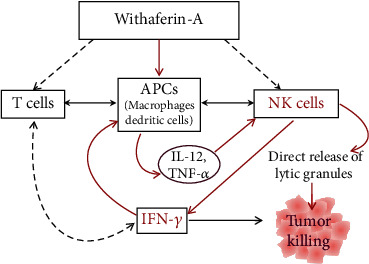
WFA triggers immunostimulatory cascade associated with NK cell regulation and antitumor immunity.

When boosting the body's immune system for the prevention or treatment of cancer, one of the critical components is to activate the antigen-presenting cells (APCs) such as macrophages and dendritic cells (DCs). Induction of Th1-type immune response is associated with tumor suppression, while Th2 immune responses are tumor-promoting. It is noticeable that WFA treatment polarized the immune response towards the Th1 type of immunity, increasing IFN-*γ* and IL-12 while decreasing Th2 type-associated IL-4, IL-10, and TGF-*β* cytokines. Thus, WFA injection of mice potentially activates APCs to release IL-12 and TNF-*α* cytokines activating NK cell functions; however, such a hypothesis needs further validation.  Figure 3 describes a schematic presentation for the potential mechanism of WFA-induced immune cell activation.

## 5. Clinical Potential of WFA

A PubMed search for clinical trials using the term “*Withania somnifera*” yielded few clinical studies testing the impact of *W. somnifera*. Most of these studies consumed root extracts of *W. somnifera* alone or combined with other therapeutic drugs. The published clinical studies demonstrated the safety profile and efficacy of root preparation in various conditions, including chronic stress and anxiety [98–100], schizophrenia [101, 102], memory and cognitive improvement [103], obsessive-compulsive disorder [104], and subclinical hypothyroidism [105]. However, clinical studies testing the impact of *W. somnifera* directly in cancer patients are minimal. In an open-label prospective nonrandomized comparative trial on 100 breast cancer patients in all stages who received chemotherapy with or without oral *W. somnifera*, the use of *W. somnifera* showed an improvement in the quality-of-life and fatigue scores [106]. A phase I study of WFA in advanced-stage high-grade osteosarcoma patients also demonstrated that WFA was safe and well-tolerated up to 4800 mg per day [107]. Surprisingly, no clinical study has tested the efficacy of WFA for prostate cancer.

## 6. Conclusions and Future Perspectives

*Withania somnifera* establishes various health benefits, including antistress, anti-inflammatory, anticancer, and immunostimulatory properties. Indeed, administration of WFA and its impact on tumor growth inhibition in TRAMP and Pten-knockout prostate cancer mouse models with functional immune systems indicate enhanced antitumor immunity. However, to further understand the role of WFA in regulating the immunobiology of prostate cancer, detailed studies are needed to analyze the infiltrating immune cells in these tumors. Many prostate cancers are organ-confined at the time of diagnosis, and some patients prefer to remain under active surveillance. Since WFA-induces Th1-type responses and NK cell activation, the slow-growing nature of some prostate cancers may provide an opportunity for immune manipulation at early prostate cancer stages and may represent a paradigm for the use of WFA for cancer-related or chemotherapy-induced fatigue, cancer prevention, and therapeutic efficacy. WFA usage combined with standard treatment regimens may help induce both innate and adaptive immune system arms to achieve greater therapeutic efficacy to target prostate cancer. The preclinical observations suggest pluripotent functions of WFA targeting prostate cancer; therefore, more extensive randomized clinical studies are needed to establish the safety profile of the WFA compound.

## Figures and Tables

**Table 1:** Nonimmune molecular targets of WFA and *Withania somnifera* (WS) root extract in prostate cancer.

Compound type	Model system	Molecular targets
Withaferin A	LNCaP, PC3, and 22RV1 cell lines	Induces cytoprotective autophagy by increasing GABARAPL1 (ATG8L) expression [11]
Withaferin A	PC3 cell line	Induction of prostate apoptosis response-4- (Par-4-) dependent apoptosis [14]
Withaferin A	PC3 and DU145 cell lines	Upregulation of Aurora B, phosphor histone H3, and phospho-Wee-1 expression and downregulation of cyclins (A2, B1, and E2) and phospho-Chk1 (Ser345), Chk2 (Thr68), and Cdc2 (Tyr15) [22]
Withaferin A	Cell lines PC3, DU145, and LNCaP	Disrupt vimentin cytoskeleton by induction of ROS and c-Fos expression and suppression of c-FLIP(L) [23]
Withaferin A	LNCaP cells and PC3 xenografts in nude mice, i.p. injection with 4 or 8 mg/kg/day for 24 days	Target *β*5 subunit and inhibition of chymotrypsin-like activity in vivo tumors of PC3 xenografts with an accumulation of proteasome target proteins (p27, Bax, and I*κ*B-*α*) and decreased AR protein in in vitro LNCaP cell line [32]
Withaferin A	TRAMP model, oral gavage of 5 mg/kg	Prevent prostate adenocarcinoma, inhibit AKT signaling, and activate Foxo3a-Par-4-induced cell death and EMT markers (vimentin, *β*-catenin, and snail and upregulate E-cadherin) [44]
Withaferin A	Pten-KO mice, oral gavage with 3 or 5 mg/kg	Inhibit primary tumor growth and lung metastasis, downregulation of pAKT-mediated EMT markers [46]
Withaferin A and ethanol extract of WS root	LNCaP and 22RV1 cell lines	Inhibits fatty acid synthesis by decreasing fatty acid metabolism enzymes: acetyl-CoA carboxylase 1, ATP citrate lyase, carnitine palmitoyltransferase 1A, and fatty acid synthase with a decrease in c-Myc and pAKT [49]
WS root extract	PC3 cell line	Inhibit expression levels of cyclooxygenase-2 and interleukin-8 [50]

AR: androgen receptor; EMT: epithelial-to-mesenchymal transition; i.p.: intraperitoneal; LPS: lipopolysaccharides; PARP: poly (ADP-ribose) polymerase; ROS: reactive oxygen species; TRAMP: transgenic adenocarcinoma of mouse prostate.

## Data Availability

The additional experimental data used to support the findings of this study are included within the article.
